# Overlapping group between non‐alcoholic fatty liver disease and metabolic associated fatty liver disease better for liver research

**DOI:** 10.1002/jgh3.70039

**Published:** 2024-10-13

**Authors:** Yu‐Ming Cheng, Tsung‐Han Hsieh, Chia‐Chi Wang, Jia‐Horng Kao

**Affiliations:** ^1^ Department of Gastroenterology and Hepatology Tung's Taichung MetroHarbor Hospital Taichung City Taiwan; ^2^ Department of Research Taipei Tzu Chi Hospital, Buddhist Tzu Chi Medical Foundation Taipei Taiwan; ^3^ Department of Gastroenterology Taipei Tzu Chi Hospital, Buddhist Tzu Chi Medical Foundation and School of Medicine, Tzu Chi University Hualien Taiwan; ^4^ Division of Gastroenterology and Hepatology, Department of Internal Medicine National Taiwan University Hospital Taipei Taiwan; ^5^ Graduate Institute of Clinical Medicine, National Taiwan University College of Medicine Taipei Taiwan

**Keywords:** metabolic associated fatty liver disease, metabolic dysfunction, non‐alcoholic fatty liver disease fibrosis score, non‐alcoholic fatty liver disease, overlapping fatty liver disease

## Abstract

**Aims:**

Metabolic associated fatty liver disease (MAFLD) was proposed to replace “non‐alcoholic fatty liver disease (NAFLD) with new diagnostic criteria.” The group meeting these two diagnostic criteria is called “Overlapping Fatty Liver Disease (FLD).” Its clinical characteristics remain unknown.

**Methods:**

This study included participants from the Taiwan Bio‐Bank database, where NAFLD was defined as hepatic steatosis in liver ultrasound, with exclusion of other known chronic liver diseases. MAFLD was defined as the presence of hepatic steatosis plus metabolic dysfunction, defined as having any of following three criteria: overweight/obesity, type 2 diabetes mellitus (DM), or ≥2 metabolic risk abnormalities in lean/normal weight subjects. According to these two diagnostic criteria, three groups were identified: “overlapping FLD”, “NAFLD alone”, and “MAFLD alone.” NAFLD fibrosis score (NFS) >0.675 was defined as advanced liver fibrosis.

**Results:**

Eight thousand thirty‐eight NAFLD participants (age 55.86 ± 10.12; males 41.07%) were included in the final analysis. Of them, “overlapping FLD” was diagnosed in 7377 (91.8%) and “NAFLD alone” in 661 (8.2%) participants. “Overlapping FLD” patients were older and had a higher percentage of male, worse metabolic profiles, higher NFS, and the percentage of carotid plaques was higher than those with “NAFLD alone.” Multivariate analysis showed age, hypertension, DM, and BMI were positively associated with advanced liver fibrosis in “overlapping FLD” patients.

**Conclusions:**

“Overlapping FLD” is better for liver research due to identifying a high‐risk population among NAFLD patients. NAFLD definition introduces the heterogeneity through “NAFLD alone” group and MAFLD criteria overcome this limitation.

## Graphical abstract

Metabolic associated fatty liver disease (MAFLD) was proposed to replace “non‐alcoholic fatty liver disease (NAFLD),” with new diagnostic criteria in 2020. Overlapping fatty liver disease of MAFLD and NAFLD is equal to pure MAFLD, defined as MAFLD without other etiologies. It has the characteristics of high homogeneity and can identify high‐risk population of advanced liver fibrosis, suggesting pure MAFLD is better for steatotic liver research.

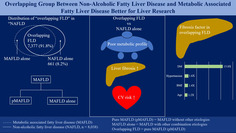

## Introduction

In 2020, the term “metabolic (dysfunction) associated fatty liver disease” (MAFLD) was proposed as a replacement for the previous term “non‐alcoholic fatty liver disease”(NAFLD).^1^ The new name includes the underlying cause of the disease and defines metabolic dysfunction (MD) in its diagnostic criteria.^2^ However, this new name does not exclude other chronic liver diseases such as those caused by viruses, alcohol, or other known causes. Although there are positive opinions about the new disease name, it has not been universally accepted. MAFLD is more understandable for patients and easier to explain for doctors in clinical practice. Because it does not exclude patients with other chronic liver diseases, it can provide more comprehensive care for MAFLD patients with other etiologies.^3^, ^4^, ^5^, ^6^ The lack of an effective drug treatment for NAFLD is the main unresolved issue in clinical practice, possibly due to the heterogeneity of the patient population. Therefore, further research is necessary to identify a disease population using better diagnostic criteria and develop a disease name that can be widely accepted, achieve the requirements of clinical practice and research, and meet the expectations of doctors and patients. Recently, there have been studies that focus on comparing the two disease names MAFLD with NAFLD. They found MAFLD covers more patients with higher liver and metabolic risks, and 80% of patients meet two‐disease diagnosis being referred to as having “overlapping fatty liver disease (FLD)” or “Dual diagnosis Fatty Liver Disease.”^7^, ^8^, ^9^, ^10^ This special group not only accounts for the majority of fatty liver patients, but also has the lowest heterogeneity. This is because, in addition to requiring metabolic dysfunction, other chronic liver diseases are also excluded. MAFLD can be divided into two groups based on the presence of other etiologies: “pure MAFLD” and MAFLD with other etiologies. The criteria of “overlapping FLD” are equal to “pure MAFLD.” This population is valuable for the development of future drugs and the exploration of liver diseases. “MAFLD alone” is the same as MAFLD with other etiologies. The “MAFLD alone” group was excluded for avoiding the confounding effects of other etiologies. The comparison of clinical characteristics between “overlapping FLD” and “NAFLD alone” groups were made using a large, population‐based Taiwan Bio‐bank database.

## Methods

### Patients and study design

The Taiwan Bio‐bank is a population‐based research database in Taiwan that has been operational since 2008, with approximately 181 635 participants enrolled through 43 recruitment stations till October 31, 2022. Data were collected using standardized procedures described in previous studies, and participants were invited for follow‐up at intervals of 2–4 years.^11^ Liver ultrasound, dual‐energy X‐ray absorptiometry (DXA), and carotid duplex ultrasound were performed in addition to the previous questionnaires and laboratory tests at the first follow‐up visit.

For this study, the participants having the first follow‐up visit were recruited. NAFLD is defined as fatty liver in liver ultrasound, with exclusion of chronic hepatitis B virus (HBV), hepatitis C virus infection, or other known causes of chronic liver disease.^12^ Participants with persistent alcohol intake >210 g/week for men and >140 g/week for women with a period of at least 3 months were also excluded from the diagnosis of NAFLD. MAFLD was diagnosed if having fatty liver plus metabolic dysfunction (MD), defined as having any of the following three criteria: overweight/obesity (body mass index [BMI] >23 kg/m^2^), type 2 diabetes mellitus (DM), or lean/normal weight, with the presence of ≥2 metabolic risk abnormalities. According to these two diagnostic criteria, the participants can be divided into three groups: “overlapping FLD”, “NAFLD alone”, and “MAFLD alone.” “MAFLD alone” group is equal to MAFLD with other etiologies. In contrast, we define “pure MAFLD” as MAFLD without other etiologies, which is equal to “overlapping FLD.” NAFLD fibrosis score (NFS) >0.675 was defined as advanced liver fibrosis and atherosclerosis was diagnosed if having plaques on carotid duplex ultrasound.^13^ In this large, population‐based study, we aim to compare the liver and atherosclerotic risks of “overlapping FLD” with “NAFLD alone” groups. In addition, the factors associated with advanced liver fibrosis in “overlapping FLD” patients were investigated.

### Ethical considerations

This study was performed in accordance with the principles of the 1975 Declaration of Helsinki and approved with waived informed consent by the Research Ethics Committee of Taipei Tzu Chi Hospital, Buddhist Tzu Chi Medical Foundation (approval numbers: 10‐XD‐055 and 11‐X‐074), and the Ethics and Governance Council of the TWB (approval numbers: TWBR11102‐03).

### Statistical analyses

**Figure 1:**
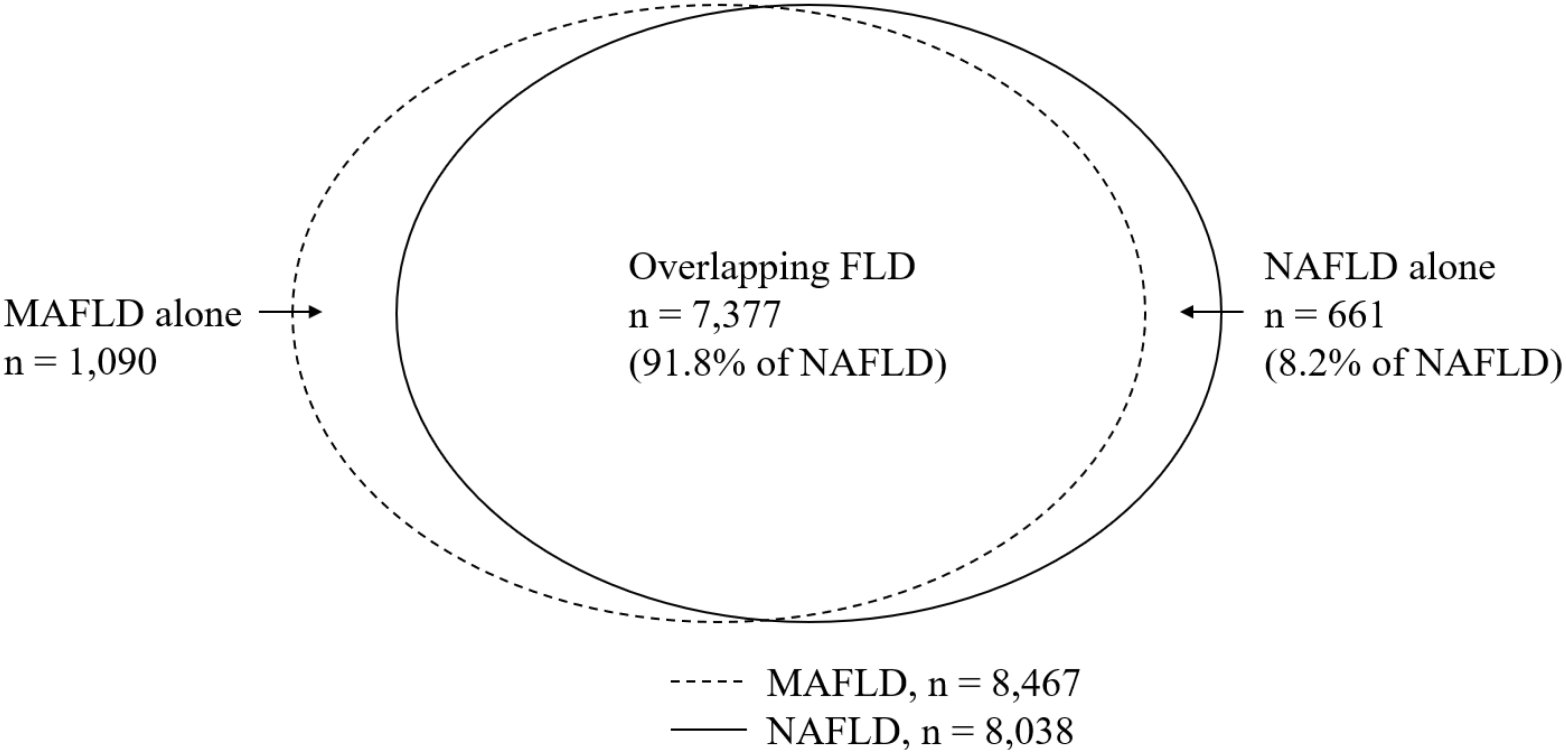
Distribution of “dual diagnosis fatty liver disease” in “non‐alcoholic fatty liver disease.” FLD, fatty liver disease; MAFLD, Metabolic associated fatty liver disease; NAFLD, non‐alcoholic fatty liver disease.

The data were expressed as mean ± standard deviation for continuous variables and number (percentage) for categorical variables. Statistical analysis was performed using SPSS version 26.0 (SPSS Inc. Chicago, IL). The clinical characteristics and outcomes were compared between the patients with “overlapping FLD” and “NAFLD alone” using propensity score matching for age and sex. These data were analyzed using chi‐square test and Student's *t* test. A *P* value less than 0.05 was considered statistically significant. The factors associated with advanced liver fibrosis in “overlapping FLD” patients were analyzed using univariate and multivariate analyses after adjustment for confounders (Fig. 1).

## Results

The study surveyed a total of 22 909 participants, with data of liver ultrasound from the Taiwan bio‐bank database. Of them, NAFLD was diagnosed in 8038 participants (age 55.86 ± 10.12; males 41.07%) and MAFLD in 8467 participants. A total of 7377 (91.8%) participants were “Overlapping FLD”, 661 (8.2%) participants were categorized as “NAFLD alone” group. In the patients with “overlapping FLD”, the proportion of elevated ALT (ALT>33 U/L in men and ALT>25 in women) is 36.1%, advanced liver fibrosis is defined as NFS >0.675 is 1.5%, cirrhosis is 0.01%, and hepatocellular carcinoma (HCC) is 0.03%. The proportion of atherosclerosis is 34.76% (Table 1).

**Table 1:** Comparison of clinical characteristics between “overlapping fatty liver disease” and “non‐alcoholic fatty liver disease alone” groups

	Overlapping FLD	NAFLD alone	*P*‐value
	*N* = 7377	*N* = 661	
Age, year	56.11 ± 10.08	53.04 ± 10.18	<0.001
Male, *N* (%)	3150 (42.70)	151 (22.84)	<0.001
BMI, kg/m^2^	26.46 ± 3.55	21.08 ± 1.44	<0.001
BMI ≥ 23 kg/m^2^, *n* (%)	6343 (86.9%)	0	<0.001
Body fat %	31.66 ± 7.48	25.39 ± 5.73	<0.001
WC, cm	89.46 ± 9.22	75.41 ± 5.93	<0.001
Metabolic parameters			
Diabetes, *N* (%)	1788 (24.24)	0	<0.001
Hypertension, *N* (%)	1824 (24.73)	31 (4.69)	<0.001
Hyperlipidemia, *N* (%)	1216 (16.48)	51 (7.72)	<0.001
Glucose, mg/dL	103.11 ± 26.00	90.46 ± 6.62	<0.001
HbA1c, %	6.15 ± 0.99	5.57 ± 0.28	<0.001
TG, mg/dL	157.74 ± 95.56	85.75 ± 35.62	<0.0001
CHO, mg/dL	199.89 ± 37.29	203.05 ± 36.21	0.036
HDL, mg/dL	48.94 ± 11.02	62.51 ± 12.80	<0.001
LDL, mg/dL	125.30 ± 33.37	123.56 ± 31.75	0.198
Liver parameters			
AST, U/L	26.96 ± 14.51	23.00 ± 7.37	<0.001
ALT, U/L	30.22 ± 26.31	19.19 ± 10.74	<0.001
Elevated ALT, *n* (%)	2665 (36.1)	94 (14.2)	<0.001
GGT, U/L	29.26 ± 29.17	17.92 ± 13.07	<0.001
Fatty liver index	42.65 ± 24.46	8.32 ± 6.76	<0.001
NFS	−1.91 ± 1.23	−2.41 ± 1.06	<0.001
NFS ≥ 0.675, *n* (%)	113 (1.5)	1 (0.2)	0.001
Other parameters			
Uric acid, mg/dL	5.91 ± 1.41	4.80 ± 1.11	<0.001
eGFR, mL/min/1.73^2^	103.33 ± 24.41	111.58 ± 23.83	0.036
Carotid plaque, *N* (%)	2564 (34.76)	141 (21.33)	<0.001
Secondary outcome			
Liver cirrhosis	1 (0.01)	0	0.916
HCC	2 (0.03)	0	0.839

*P*s. Elevated ALT is defined as ALT >33 U/L in men or ALT >25 U/L in women.

ALT, alanine aminotransferase; AST, aspartate aminotransferase; BMI, body mass index; eGFR, estimated glomerular filtration rate; FLD, fatty liver disease; GGT, γ‐glutamyl transferase; HbA1c, glycated hemoglobin; HCC, hepatocellular carcinoma; HDL, high‐density lipoprotein; LDL, low‐density lipoprotein; NAFLD, non‐alcoholic fatty liver disease; NFS, NAFLD fibrosis score; TG, triglyceride; WC, waist circumference.

### Comparison of clinical characteristics and outcomes between participants with “overlapping FLD” and “NAFLD alone”

Compared with the participants of “NAFLD alone”, “overlapping FLD” patients were older and had a higher percentage of males, DM, hypertension history, and hyperlipidemia history, higher levels of body mass index (BMI), body fat, waist circumference (WC), glucose, HbA1C, triglyceride, aspartate aminotransferase (AST), alanine aminotransferase (ALT), gamma‐glutamyl transferase (GGT), FLI, NFS, uric acid, and the percentage of carotid plaques, but lower levels of cholesterol, HDL, and estimated glomerular filtration rate (eGFR). The level of LDL was comparable between two groups (Table 1).

### Comparison of clinical characteristics and outcomes between participants with “overlapping FLD” and “NAFLD alone” using propensity score matching for age and sex (Table 2)

**Table 2:** Comparison of clinical characteristics between “overlapping fatty liver disease” and “non‐alcoholic fatty liver disease alone” groups using propensity score matching for age and sex

	Overlapping FLD (*N* = 661)	NAFLD alone (*N* = 661)	*P*‐value
Age, year	53.04 ± 10.18	53.04 ± 10.18	>0.999
Male, *N* (%)	151 (22.84)	151 (22.84)	>0.999
BMI, kg/m^2^	26.49 ± 3.89	21.08 ± 1.44	<0.001
Body fat %	34.12 ± 7.18	25.39 ± 5.73	<0.001
WC, cm	88.27 ± 9.48	75.41 ± 5.93	<0.001
Metabolic parameters			
Diabetes, *N* (%)	141 (21.33)	0	<0.001
Hypertension, *N* (%)	143 (21.63)	31 (4.69)	<0.001
Hyperlipidemia, *N* (%)	87 (13.16)	51 (7.72)	0.001
Glucose, mg/dL	101.89 ± 28.60	90.46 ± 6.62	<0.001
HbA1c, %	6.09 ± 0.93	5.57 ± 0.28	<0.001
TG, mg/dL	157.27 ± 92.55	85.75 ± 35.62	<0.001
CHO, mg/dL	201.63 ± 37.05	203.05 ± 36.21	0.480
HDL, mg/dL	49.49 ± 10.87	62.51 ± 12.80	<0.001
LDL, mg/dL	126.98 ± 33.11	123.56 ± 31.75	0.055
Liver parameters			
AST, U/L	26.27 ± 11.78	23.00 ± 7.37	<0.001
ALT, U/L	29.85 ± 27.45	19.19 ± 10.74	<0.001
GGT, U/L	29.50 ± 52.80	17.92 ± 13.07	<0.001
Fatty liver index	40.96 ± 25.18	8.32 ± 6.76	<0.001
NFS	−2.19 ± 1.20	−2.41 ± 1.06	0.005
Other parameters			
Uric acid, mg/dL	5.70 ± 1.37	4.80 ± 1.11	<0.001
eGFR, mL/min/1.73^2^	108.14 ± 25.56	111.58 ± 23.83	0.012
Carotid plaque, *N* (%)	192 (29.05)	141 (21.33)	0.001

ALT, alanine aminotransferase; AST, aspartate aminotransferase; BMI, body mass index; eGFR, estimated glomerular filtration rate; FLD, fatty liver disease; GGT, γ‐glutamyl transferase; HbA1c, glycated hemoglobin; HDL, high‐density lipoprotein; LDL, low‐density lipoprotein; NAFLD, non‐alcoholic fatty liver disease; NFS, NAFLD fibrosis score; TG, triglyceride; WC, waist circumference.

Compared with the participants of “NAFLD alone”, “overlapping FLD” patients had a higher percentage of DM, hypertension history, and hyperlipidemia history, higher levels of BMI, body fat, WC, glucose, HbA1C, triglyceride, AST, ALT, GGT, FLI, NFS, and uric acid, but a lower level of eGFR. The levels of cholesterol and LDL were comparable between two groups. The percentage of carotid plaque in the “overlapping FLD” patients was higher than that in “NAFLD alone” patients (*P* = 0.001).

### Comparison of the clinical outcomes between “NAFLD alone” group and healthy controls

The healthy controls are defined as having neither fatty liver nor metabolic dysfunction. Compared with the healthy controls, patients with “NAFLD alone” are older and have higher BMI, WC, glucose, HbA1c, triglycerides, total cholesterol, LDL, ALT, GGT, FLI, and higher percentages of hypertension, hyperlipidemia, and carotid plaques. There is no difference in terms of NFS, AST, and gender proportions between the two groups (Table 3).

**Table 3:** Comparison of clinical characteristics between “non‐alcoholic fatty liver disease alone” and “healthy control” groups

	NAFLD alone	Healthy control	*P*‐value
	*N* = 661	*N* = 2182	
Age, year	53.04 ± 10.18	48.74 ± 9.98	<0.001
Male, *N* (%)	151 (22.84)	414 (19.0)	0.077
BMI, kg/m^2^	21.08 ± 1.44	20.42 ± 1.58	<0.001
Body fat %	25.39 ± 5.73	24.12 ± 5.58	<0.001
WC, cm	75.41 ± 5.93	72.74 ± 5.39	<0.001
Metabolic parameters			
Diabetes, *N* (%)	0	0	–
Hypertension, *N* (%)	31 (4.69)	0	<0.001
Hyperlipidemia, *N* (%)	51 (7.72)	0	<0.001
Glucose, mg/dL	90.46 ± 6.62	87.44 ± 5.20	<0.001
HbA1c, %	5.57 ± 0.28	5.38 ± 0.21	<0.001
TG, mg/dL	85.75 ± 35.62	68.48 ± 25.12	<0.001
CHO, mg/dL	203.05 ± 36.21	192.73 ± 32.54	<0.001
HDL, mg/dL	62.51 ± 12.80	66.17 ± 11.83	<0.001
LDL, mg/dL	123.56 ± 31.75	111.15 ± 28.45	<0.001
Liver parameters			
AST, U/L	23.00 ± 7.37	23.19 ± 8.39	0.620
ALT, U/L	19.19 ± 10.74	16.88 ± 12.97	<0.001
GGT, U/L	17.92 ± 13.07	15.12 ± 12.35	<0.001
Fatty liver index	8.32 ± 6.76	4.78 ± 3.87	<0.001
NFS	−2.41 ± 1.06	−2.35 ± 1.00	0.197
NFS ≥ 0.675, *n* (%)	1 (0.2)	3 (0.1)	0.934
Other parameters			
Uric acid, mg/dL	4.80 ± 1.11	4.51 ± 1.04	<0.001
eGFR, mL/min/1.73^2^	111.58 ± 23.83	113.73 ± 22.95	0.037
Carotid plaque, *N* (%)	141 (21.33)	275 (12.6)	<0.001
Secondary outcome			
Liver cirrhosis	0	0	
HCC	0	0	

ALT, alanine aminotransferase; AST, aspartate aminotransferase; BMI, body mass index; eGFR, estimated glomerular filtration rate; GGT, γ‐glutamyl transferase; HbA1c, glycated hemoglobin; HCC, hepatocellular carcinoma; HDL, high‐density lipoprotein; LDL, low‐density lipoprotein; NAFLD, non‐alcoholic fatty liver disease; NFS, NAFLD fibrosis score; TG, triglyceride; WC, waist circumference.

### Comparison of the clinical characteristics between MAFLD and NAFLD

Compared with patients with NAFLD, those with MAFLD have a higher proportion of males and diabetes. They also have higher BMI, WC, body fat, glucose, HbA1c, triglycerides, liver inflammation markers, and NFS, but lower HDL levels. The age, total cholesterol, LDL, and the proportion of carotid plaques are similar between the two groups (Table 4).

**Table 4:** Comparison of clinical characteristics between “metabolic associated fatty liver disease” and “non‐alcoholic fatty liver disease” groups

	MAFLD (*N* = 8467)	NAFLD (*N* = 8038)	*P*‐value
Age, year	55.93 ± 10.02	55.86 ± 10.12	0.655
Male, *N* (%)	3734 (44.1)	3301 (41.1)	0.012
BMI, kg/m^2^	26.49 ± 3.54	26.02 ± 3.73	<0.001
Body fat %	31.54 ± 7.48	31.14 ± 7.55	0.001
WC, cm	89.57 ± 9.23	88.31 ± 9.79	<0.001
Metabolic parameters			
Diabetes, *N* (%)	2047 (24.2)	1788 (22.2)	0.020
Hypertension, *N* (%)	2086 (24.6)	1855 (23.1)	0.066
Hyperlipidemia, *N* (%)	1372 (16.2)	1267 (15.8)	0.510
Glucose, mg/dL	103.21 ± 26.05	102.07 ± 25.22	0.004
HbA1c, %	6.14 ± 1.00	6.10 ± 0.97	0.009
TG, mg/dL	157.38 ± 96.94	151.82 ± 94.21	<0.001
CHO, mg/dL	199.30 ± 37.35	200 ± 37.21	0.143
HDL, mg/dL	48.92 ± 11.06	50.06 ± 11.78	<0.001
LDL, mg/dL	124.77 ± 33.38	125.15 ± 33.24	0.464
Liver parameters			
AST, U/L	27.33 ± 15.06	26.40 ± 14.10	0.002
ALT, U/L	30.84 ± 29.61	29.31 ± 25.57	<0.001
GGT, U/L	29.88 ± 32.19	28.33 ± 28.36	0.001
Fatty liver index	42.85 ± 24.59	39.83 ± 25.33	<0.001
NFS	−1.90 ± 1.23	−1.95 ± 1.23	<0.001
Other parameters			
Uric acid, mg/dL	5.92 ± 1.41	5.82 ± 1.42	<0.001
eGFR, mL/min/1.73^2^	103.37 ± 24.29	104.0 ± 24.46	0.097
Carotid plaque, *N* (%)	2906 (34.3)	2705 (33.7)	0.525

ALT, alanine aminotransferase; AST, aspartate aminotransferase; BMI, body mass index; eGFR, estimated glomerular filtration rate; GGT, γ‐glutamyl transferase; HbA1c, glycated hemoglobin; HDL, high‐density lipoprotein; LDL, low‐density lipoprotein; MAFLD, metabolic associated fatty liver disease; NAFLD, non‐alcoholic fatty liver disease; NFS, NAFLD fibrosis score; TG, triglyceride; WC, waist circumference.

### Factors associated with advanced liver fibrosis in patients with “overlapping FLD” using univariate and multivariate analysis

Univariate analysis showed age, DM, HT, hyperlipidemia, BMI, and FLI were associated with advanced liver fibrosis. After adjusting for confounders, multivariate analysis showed that age, DM, HT, and BMI were positively associated with advanced liver fibrosis in the “overlapping FLD” patients, but a negative association with FLI (Table 5).

**Table 5:** Factors associated with advanced liver fibrosis, based on NFS > 0.675 using univariate and multivariate analyses in patients with “overlapping fatty liver disease“ (*n* = 7377)

	Univariate analysis			Multivariate analysis		
NFS >0.675	OR	95% CI	*P* value	AOR	95% CI	*P* value
Age	1.17	1.13–1.20	<0.001	1.18	1.14–1.23	<0.001
Male	1.07	0.73–1.55	0.738	1.04	0.69–1.57	0.866
DM	21.55	12.48–37.20	<0.001	15.84	8.95–28.04	<0.001
HTN	5.11	3.49–7.51	<0.001	1.57	1.02–2.41	0.042
Hyperlipidemia	2.03	1.34–3.07	0.001	0.77	0.48–1.23	0.274
BMI, kg/m^2^	1.19	1.15–1.24	<0.001	1.38	1.27–1.50	<0.001
GGT, U/L	1.002	0.998–1.006	0.424	1.003	0.996–1.009	0.404
Fatty liver index	1.02	1.02–1.03	<0.001	0.98	0.947–0.996	0.015

Level of significance: *P* < 0.05 (binary logistic regression).

AOR, adjusted odds ratio; BMI, body mass index; CI, confidence interval; DM, diabetes mellitus; GGT, γ‐glutamyl transferase; HTN, hypertension; NFS, NAFLD fibrosis score; OR, odds ratio.

## Discussion

In the large population‐based study, NAFLD and MAFLD were diagnosed in 8038 and 8467 participants, respectively. Among NAFLD patients, “overlapping FLD” was found in 7377 (91.8%) participants and “NAFLD alone” in 661 (8.2%) participants. Compared with “NAFLD alone” group, participants in “Overlapping FLD” group were older and had a higher percentage of male. For avoiding their confounding effects, propensity score matching for age and sex consistently showed worse metabolic profiles, higher NFS, and higher percentage of carotid plaques in “overlapping FLD.” Furthermore, multivariate analysis showed age, DM, and BMI were associated with advanced liver fibrosis in patients with “overlapping FLD.” In addition, NAFLD definition introduces the heterogeneity through “NAFLD alone” group and MAFLD criteria overcome this limitation.

In 2020, the term “MAFLD” was proposed to replace the previous name of “NAFLD.” However, this new name has not been universally accepted by all societies. The change in name is not the main reason for the controversy, but rather the change in diagnostic criteria. The new diagnosis includes the criteria of MD to fit the new name, and other chronic liver diseases are not excluded. This change in diagnostic criteria may lead to differences in the population affected, and in turn changes in the natural history and long‐term prognosis of the disease. Recent studies have shown that the new disease name can clinically include more patients and identify those with higher disease severity.^14^, ^15^, ^16^ Our study also shows higher proportion of diabetes, worse metabolic profiles, and higher severity of hepatic outcomes in patients with MAFLD than those with NAFLD. From a holistic perspective of healthcare, NAFLD not only affects the liver, but can also lead to the development of extra‐hepatic complications such as DM or cardiovascular disease (CVD), etc.^17^, ^18^ If the patients with other chronic liver diseases such as viral, alcoholic, etc., are excluded in the situation of NAFLD, these patients may not receive comprehensive care.^19^ However, from a liver disease research perspective, the best inclusion criteria should include the diagnostic criteria of MD while excluding the patients with other causes of chronic liver diseases. This population is the same as the group of “overlapping FLD”, meaning that they simultaneously meet the criteria of NAFLD and MAFLD. The study showed that the “overlapping FLD” group accounted for 91.9% of NAFLD patients and had a higher disease severity, including a greater risk of liver fibrosis and atherosclerosis. These findings were also confirmed after propensity score matching for age and sex. Therefore, this group requires more attention and treatment in clinical settings. The study's findings suggest that clinical research of MAFLD for liver outcomes or drug development should include “overlapping FLD” patients, who are equal to “pure MAFLD” patients.^20^

The main difference between “overlapping FLD” and “NAFLD alone” groups is the presence of MD. Liver fibrosis itself is related to the prognosis and liver outcomes including the development of cirrhosis, end‐stage liver disease, or hepatocellular carcinoma in NAFLD patients. Previous research has shown that diabetic and obese patients have a higher risk of developing fibrosis, liver cirrhosis, and hepatocellular carcinoma.^21^, ^22^, ^23^, ^24^ This study shows NAFLD patients with MD, equal to “overlapping FLD”, have a higher risk of advanced liver fibrosis and atherosclerosis than those without. Compared with health controls, “NAFLD alone” patients had worse metabolic profiles and higher risk of atherosclerosis. Regarding the liver outcomes, the “NAFLD alone” patients had higher liver inflammatory markers than healthy controls, but there was no difference in the proportions of advanced liver fibrosis, cirrhosis, and HCC between these two groups. Additionally, the study also indicates that age, DM, and BMI were associated with advanced liver fibrosis in “overlapping FLD” patients, suggesting MD by itself or indirectly through hepatic steatosis can increase the severity of liver fibrosis.

This study has strengths and weaknesses. The strengths include: (i) As far as I know, this is the first study to investigate the clinical characteristics of “overlapping FLD” patients, which meet two diagnostic criteria of NAFLD and MAFLD. (ii) The large‐scale population‐based study confirms that the clinical severity of this group is higher in NAFLD patients. (iii) Our study first recommends enrolling the patients with the criteria of “overlapping FLD” for the study of liver research. Some of the weaknesses in this study are as follows. (i) The diagnosis of fatty liver is mainly based on liver ultrasound rather than biopsy. However, biopsy is a more invasive examination and is not well suited for general population studies. (ii) Diagnosis of fatty liver by ultrasound is not sensitive for patients with mild fatty liver under histology.^25^ (iii) Because it is a cross‐sectional study, the causal relationship between metabolic components and liver fibrosis cannot be confirmed. (iv) There are no data about homeostasis model assessment‐insulin resistance index (HOMA‐IR), hs‐CRP, and details for excluding autoimmune diseases in our study. Furthermore, there are no participants with the data of fibroscan in the population‐based database.

In summary, this large population‐based study found that “overlapping FLD” contained 91.9% of NAFLD patients and better identified a high‐risk population of advanced liver fibrosis and atherosclerosis. Multivariate analysis revealed that age, DM, and BMI were associated with advanced liver fibrosis in patients with “overlapping FLD”, suggesting the studies of MAFLD for liver outcomes should select “overlapping FLD” group, which is equal to the definition of “pure MAFLD.” Because this approach excludes patients with other causes of liver diseases and includes those with greater disease severity and the highest treatment needs, it can provide significant aid for future liver research studies, especially for drug development.

In clinical practice, we often encounter patients with chronic hepatitis B or hepatitis C virus infection. Some of them have already undergone treatment, and the viruses have been controlled or even completely cleared. However, if they have evidence of fatty liver on imaging or pathology, these patients would be excluded from the diagnosis of NAFLD. As a result, they may not receive the recommended monitoring and care. The diagnostic criteria for MAFLD provide more comprehensive care for these patients in clinical practice. However, when conducting studies of liver research, “pure MAFLD” is better for patient selection. This approach allows for a higher degree of patient homogeneity, which can greatly assist in liver research outcomes and the development of future treatments.

## Data Availability

We declare the data were available and approved by the Ethics and Governance Council of the TWB (approval numbers: TWBR11102‐03).
